# Reliable Pseudo-Labeling and Confusion Calibration for Foggy-Scene Semantic Segmentation

**DOI:** 10.3390/jimaging12070289

**Published:** 2026-06-30

**Authors:** Shuai Yan, Shirong Feng, Zhicheng Wei

**Affiliations:** 1 College of Computer and Cyber Security, Hebei Normal University, Shijiazhuang 050024, China; yanshuai@stu.hebtu.edu.cn; 2 Key Lab of Network and Information Security of Hebei Province, Shijiazhuang 050024, China; 3 Hebei Engineering Research Center for Supply Chain Big Data Analysis and Data Security, Shijiazhuang 050024, China

**Keywords:** unsupervised domain adaptation, semantic segmentation, foggy scenes, pseudo-label, energy-based learning, class confusion

## Abstract

Semantic segmentation in foggy scenes is crucial for autonomous driving systems, yet acquiring annotated real-world foggy data is highly costly. Existing unsupervised domain adaptation methods typically adopt a self-training strategy, adapting models trained under clear-weather conditions to unlabeled foggy target domains and constructing supervision signals via pseudo-labels. However, these methods mainly focus on improving the reliability of target-domain supervision while paying insufficient attention to class confusion caused by the degradation of class discriminability. In fact, the performance degradation of self-training in foggy scenarios is not caused by a single factor, but is jointly affected by unreliable supervision signals and reduced class discriminability. To address these issues, this paper proposes a reliable pseudo-labeling and confusion calibration framework for foggy-scene semantic segmentation, termed RPCC. Specifically, dynamic energy-guided pseudo-labeling (DEPL) models the reliability of target-domain predictions using energy scores, thereby improving the reliability of target-domain supervision signals. Furthermore, the reliable-region class confusion calibration (RCC) module models and calibrates semantic class relationships in target-domain predictions based on reliable pseudo-label supervision, thereby suppressing class confusion and enhancing semantic boundary clarity. Experimental results demonstrate that RPCC outperforms existing methods on multiple real-world foggy-scene datasets and shows favorable generalization to other adverse weather conditions.

## 1. Introduction

Semantic segmentation aims to assign semantic category labels to each pixel in an image and is one of the key techniques in visual tasks such as autonomous driving, intelligent transportation, and scene understanding. In recent years, deep neural networks have improved semantic segmentation performance. However, existing semantic segmentation models usually rely on data collected and annotated under clear-weather conditions for training. When these models are directly applied to real-world foggy scenes, their segmentation performance often decreases significantly due to reduced visibility, blurred local structures, and weakened texture details. Especially near the category boundaries of roads, vehicles, pedestrians, and buildings, haze weakens the discriminative features in images, making adjacent categories or visually similar categories more likely to be confused. Therefore, improving segmentation performance in foggy scenes without real-world foggy pixel-level annotations remains a challenging problem.

To alleviate the scarcity of annotated real-world foggy data, unsupervised domain adaptation methods [1,2,3,4,5] have been proposed. These methods usually reduce the domain gap between the source domain and the target domain through image translation, feature alignment, style transfer, or self-training [6,7,8,9,10,11,12]. Among them, self-training methods construct target-domain supervision signals by generating pseudo-labels for target-domain images, and do not require additional intermediate-domain datasets or complex multi-stage training procedures, thus showing good application potential in foggy-scene domain adaptation. However, haze degradation reduces the class discriminability of target-domain images, resulting in model predictions inevitably containing errors and thus introducing noisy pseudo-labels.

To reduce the influence of noisy pseudo-labels on target-domain training, many self-training methods [4,5,6,7,8,12,13] adopt softmax confidence scores to select reliable pixels. However, this strategy still has two limitations. First, high-confidence predictions do not necessarily correspond to reliable predictions, and the model may still misidentify some incorrect pixels as reliable pseudo-labels, thereby introducing noisy supervision. Second, pseudo-label reliability is not equivalent to class separability. In real-world foggy scenes, even if some pixels are retained through reliability filtering, the prediction probabilities of adjacent categories or visually similar categories may still overlap, thereby leading to blurred class boundaries and semantic class confusion. Therefore, the degradation of self-training performance in foggy scenes is not only caused by pseudo-label noise, but is also closely related to disordered class relationships in target-domain predictions.

Based on the above analysis, this paper proposes reliable pseudo-labeling and confusion calibration (RPCC), a self-training framework for semantic segmentation in foggy scenes. RPCC optimizes target-domain self-training from two complementary aspects: pseudo-label reliability modeling and class confusion calibration. Specifically, dynamic energy-guided pseudo-labeling (DEPL) uses pixel-level energy scores to construct reliable target-domain pseudo-label supervision to reduce the influence of noisy pseudo-labels; reliable-region class confusion calibration (RCC) models class relationships within reliable prediction regions to suppress unreasonable overlaps between different class prediction distributions. Through the collaborative effect of the two components, RPCC can simultaneously alleviate noisy supervision and class confusion, thereby improving the semantic segmentation performance of the model in foggy scenes.

The main contributions of this paper are summarized as follows:

1. We propose RPCC, a reliable pseudo-labeling and confusion calibration self-training framework. Unlike self-training methods that mainly focus on pseudo-label selection, this paper attributes the degradation of self-training in foggy scenes to the joint effect of pseudo-label noise and class confusion, and collaboratively optimizes target-domain learning from the perspectives of reliable supervision construction and class confusion calibration.
2. We propose dynamic energy-guided pseudo-labeling (DEPL), which uses energy scores to construct reliable target-domain pseudo-label supervision and improves the stability of pseudo-label supervision through training-stage weight adjustment.
3. We propose reliable-region class confusion calibration (RCC), which models class relationships within reliable prediction regions and alleviates class confusion in foggy scenes through class channel normalization and off-diagonal confusion suppression.
4. The proposed RPCC outperforms existing methods on multiple real-world foggy-scene datasets and shows good generalization ability in rainy and snowy scenes.

## 2. Related Work

### Unsupervised Domain Adaptation for Semantic Segmentation in Foggy Scenes

Unsupervised domain adaptation (UDA) aims to improve model performance in the target domain by using labeled source-domain data and unlabeled target-domain data. To reduce the distribution discrepancy between the source and target domains, existing methods have been widely studied from the perspectives of image style transfer, frequency-domain transformation, normalization mechanisms, and feature constraints [3,14,15,16,17,18]. For example, FDA [3] reduces image-level domain discrepancy by exchanging the low-frequency components of source- and target-domain images. TransNorm [15] designs a transferable normalization mechanism to enhance the domain adaptation ability of the model. BSP [16], AFN [17], and TAT [18] improve cross-domain performance from the perspectives of feature transferability, feature norms, and adversarial training, respectively. These methods can reduce the distribution discrepancy between domains to a certain extent, but they mainly focus on global alignment at the image or feature level, while the use of potential supervision signals in the target domain remains limited.

To further exploit unlabeled target-domain data, self-training has gradually become an important direction in UDA semantic segmentation [2,3,6,7,8,9,10,11,12,19,20,21]. Such methods generate pseudo-labels from model predictions on the target domain and use them as supervision signals to continuously optimize the network. CBST [8] alleviates the class imbalance problem of pseudo-labels through a class-balanced strategy. CRST [7] introduces confidence regularization to improve pseudo-label quality. TPLD [6] adopts a two-phase pseudo-label densification strategy to gradually expand reliable pseudo-label regions. Subsequently, ProDA [9], DAFormer [10], MIC [11], and PL-SR [12] further improve self-training from the perspectives of prototype alignment, network architecture improvement, consistency constraint, and pseudo-label self-refinement. Although these methods improve the usability of target-domain pseudo-labels, most of them mainly focus on pseudo-label selection, confidence correction, and noise suppression, while insufficiently considering visual degradation and class confusion in real foggy scenes.

When UDA is applied to semantic segmentation from clear-weather source domains to real foggy target domains, the visual degradation caused by fog, such as reduced visibility, weakened texture, and blurred boundaries, further increases the difficulty of adaptation. Therefore, traditional semantic segmentation methods [1,22] often perform poorly in foggy environments. Early methods usually use synthetic foggy data or intermediate fog domains to alleviate the lack of annotations in real foggy scenes. However, there is still an obvious domain shift between synthetic fog and real fog, which limits their performance in real foggy scenes. To reduce this gap, CMAda3+ [20] gradually transfers the model from synthetic light-fog data to real dense-fog data through curriculum learning. FIFO [23] models the fog formation process using fog pass filtering. CuDA-Net+ [24] decomposes the domain discrepancy into style discrepancy and fog-density discrepancy, and alleviates accumulated domain shift through multi-stage curriculum training. These methods can more specifically handle domain discrepancies in foggy scenes, but they usually rely on synthetic data, intermediate-domain construction, or multi-stage training, making the practical application process relatively complex.

To reduce the dependence on additional intermediate domains and complex training procedures, some studies directly design self-training strategies for real foggy scenes. TDo-Dif [4] enhances temporal consistency through image fusion to improve the stability of target-domain features. CLD [5] reduces confusion between visually similar categories through pixel-wise candidate label disambiguation. DAEN [25] improves adaptation performance in real foggy scenes through intermediate-domain feature construction and pseudo-label optimization. These methods indicate that constructing reliable target-domain supervision signals is crucial for self-training in foggy scenes. However, methods that mainly improve pseudo-label reliability still cannot fully address class confusion caused by unclear class boundaries and overlapping probabilities of similar categories in real foggy images. Different from DAEN, which relies on intermediate-domain feature construction and pseudo-label optimization, RPCC jointly addresses pseudo-label noise and class confusion through reliable pseudo-label construction and reliable-region class confusion calibration.

In addition, some studies have begun to enhance the class discriminability of models from the perspectives of structural information, class relationships, or cross-domain semantic consistency. FreD [26] improves segmentation performance in foggy scenes by decoupling high-frequency and low-frequency information. UniDA-SS [27] uses prototype learning and distribution alignment to improve adaptation under category-mismatched conditions. ShiftMatch [28] matches feature distribution differences between the source and target domains through contrastive learning to enhance cross-domain semantic consistency. These methods improve the model’s ability to distinguish semantic categories from different perspectives, but most of them still focus on structural information utilization, prototype alignment, or feature consistency modeling, while paying insufficient attention to the interaction between pseudo-label noise and class confusion.

In summary, existing methods have promoted the development of foggy-scene semantic segmentation from the perspectives of inter-domain distribution alignment, curriculum learning, fog modeling, pseudo-label optimization, and semantic consistency enhancement. However, in real foggy scenes, pseudo-label noise weakens the reliability of target-domain supervision signals, while class confusion affects the model’s ability to distinguish adjacent and visually similar categories. These two problems often jointly limit model performance. To address this issue, this paper proposes RPCC, which collaboratively optimizes the self-training process in foggy scenes from two aspects—reliable pseudo-label construction and reliable-region class confusion calibration—thereby simultaneously alleviating noisy supervision and class confusion.

## 3. Materials and Methods

### 3.1. Motivation and Framework Overview

In the unsupervised domain adaptation task for semantic segmentation from clear weather to real foggy scenes, the source-domain samples and their semantic labels are denoted as $X_{cw}$ and $Y_{cw}$, respectively, while the unlabeled target-domain samples are denoted as $X_{rf}$. Since target-domain supervision is constructed from model predictions, the amount of semantic information preserved in $X_{rf}$ directly affects both pseudo-label reliability and class discriminability. Therefore, before presenting the proposed framework, we first analyze how foggy degradation changes the semantic information available for target-domain prediction.

To avoid assuming paired source–target samples, we introduce a latent clear-view image $X^{★}$ for each target-domain scene and denote its associated semantic random variable as Y. Here, $X^{★}$ denotes an unobserved ideal clear-view observation of the same target scene and is distinct from the observed source-domain samples $X_{cw}$. We use an idealized fog-degradation model in which the foggy observation $X_{rf}$ is generated from $X^{★}$. This model assumes that fog changes the visual observation while preserving the underlying scene semantics and that, given $X^{★}$, the fog-degradation process does not provide additional information about Y. Accordingly, the Markov relation $Y\to X^{★}\to X_{rf}$ is used only as a conceptual motivation. Under this model, the data processing inequality gives

(1) $$I\left(Y;X_{rf}\right)\leq I\left(Y;X^{★}\right),$$

where I(·;·) denotes mutual information. Importantly, this relation does not require a one-to-one correspondence between the observed source-domain samples $X_{cw}$ and target-domain samples $X_{rf}$. In the actual UDA setting, $X_{cw}$ and $X_{rf}$ are unpaired samples from different domains; no paired clear–foggy images are constructed, and no mutual information quantity is estimated or optimized during training.

Under this latent observation model, mutual information can be written as

(2) $$I\left(Y;X_{rf}\right)=H\left(Y\right)-H\left(Y|X_{rf}\right),$$

where $H(Y|X_{rf})$ denotes the semantic uncertainty given the foggy observation. For a fixed semantic prior, a decrease in $I(Y;X_{rf})$ corresponds to an increase in $H(Y|X_{rf})$. This provides an information-theoretic explanation for why fog degradation can increase prediction uncertainty. In semantic segmentation, such uncertainty may manifest as overlapping class probability distributions in boundary regions, distant objects, and low-contrast regions, thereby causing class confusion. Therefore, target-domain self-training in foggy scenes requires not only reliable pseudo-label supervision but also constraints on class confusion in target-domain predictions.

**Figure 1:**
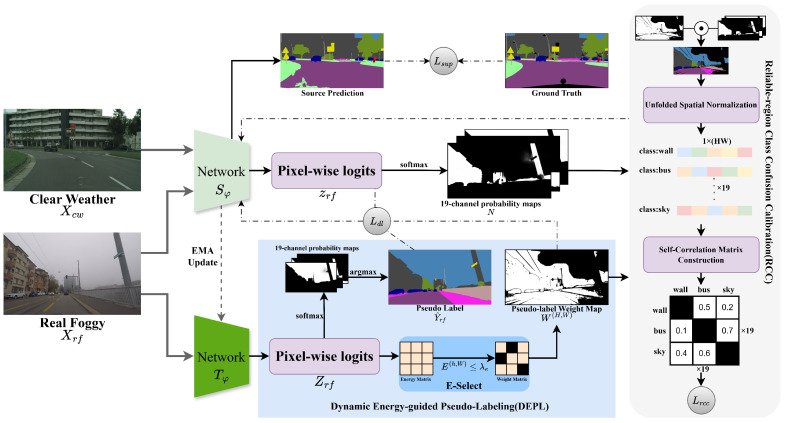
Overall framework of RPCC.

Based on the above analysis, we construct RPCC as shown in Figure 1. For source-domain samples, $S_{\varphi }$ is optimized with $L_{sup}$ using $\left(X_{cw},Y_{cw}\right)$. For target-domain samples, $X_{rf}$ is fed into $S_{\varphi }$ and its exponential moving average (EMA) branch $T_{\varphi }$. DEPL constructs the pseudo-label ${\hat{Y}}_{rf}$ and weight map *W* from $T_{\varphi }$ predictions to supervise $S_{\varphi }$, while RCC uses the probability map *N* and *W* to constrain class relationships in reliable regions. During training, $T_{\varphi }$ is updated from $S_{\varphi }$ via EMA, and RPCC is optimized by $L_{sup}$, $L_{dl}$, and $L_{rcc}$.

### 3.2. Dynamic Energy-Guided Pseudo-Labeling

In unsupervised domain adaptation training, the target domain lacks manual annotations, and thus the model usually relies on pseudo-labels to construct target-domain supervision signals. The quality of pseudo-labels directly affects the effectiveness and stability of the self-training process. Existing methods commonly use softmax confidence scores to select pseudo-labels [4,5]. However, in real foggy scenes, the model may assign high confidence to incorrect predictions, causing some erroneous pixels to be mistakenly selected as reliable pseudo-labels and thus interfering with target-domain learning.

**Figure 2:**
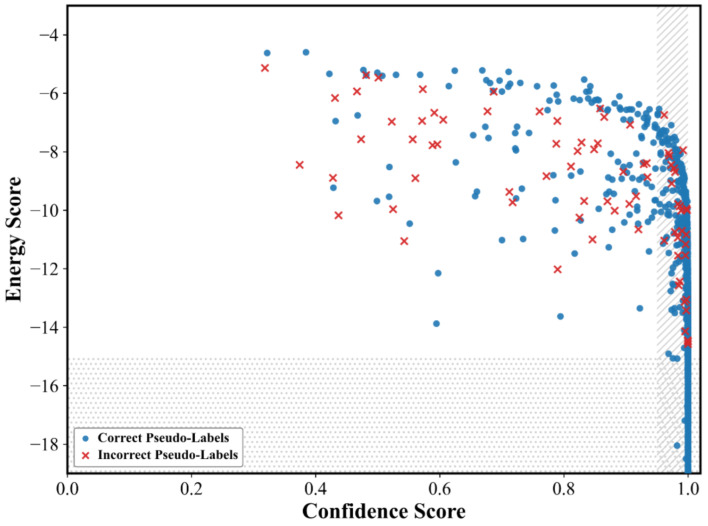
Comparison of confidence scores and energy scores for pseudo-label reliability estimation. The horizontal axis denotes the softmax confidence score, and the vertical axis denotes the pixel-level energy score. Blue dots represent correct pseudo-labels, while red crosses represent incorrect pseudo-labels. The shaded region on the right denotes the high-confidence pseudo-label region selected by a confidence threshold, and the dotted shaded region at the bottom denotes the low-energy pseudo-label region selected by an energy threshold.

Figure 2 illustrates the distributions of correct and incorrect pseudo-labels in the two-dimensional space of confidence score and energy score, where the horizontal axis denotes the softmax confidence score, and the vertical axis denotes the pixel-level energy score. Blue dots represent correct pseudo-labels, while red crosses represent incorrect pseudo-labels. The shaded region on the right denotes the high-confidence pseudo-label region selected by a confidence threshold, and the dotted shaded region at the bottom denotes the low-energy pseudo-label region selected by an energy threshold. It can be observed that some incorrect pseudo-labels are also concentrated in the high-confidence region, indicating that relying solely on confidence scores may easily misclassify incorrect predictions as reliable pseudo-labels. In contrast, no incorrect pseudo-labels are observed in the low-energy region, indicating that energy scores can more effectively distinguish reliable predictions from erroneous ones and provide a basis for constructing stable target-domain supervision signals.

Based on the above analysis, this paper proposes dynamic energy-guided pseudo-labeling (DEPL) to construct target-domain supervision signals. Given an unlabeled real foggy image $X_{rf}$, when it is fed into the network $T_{\varphi }$, the network outputs the corresponding pixel-level logits map $Z_{rf}=T_{\varphi }\left(X_{rf}\right)\in R^{C\times H\times W}$. For each pixel location (h,w), its pixel-level energy score is calculated as

(3) $$E_{X_{rf}}^{\left(h,w\right)}=-\log\left(\sum_{c=1}^{C}e^{Z_{rf}^{\left(c,h,w\right)}}\right),$$

where *c* denotes the class index; *C*, *H*, and *W* denote the number of classes, image height, and image width, respectively; and $E_{X_{rf}}^{\left(h,w\right)}$ denotes the energy score at pixel location (h,w).

Previous studies have shown that energy scores can characterize the proximity between samples and known distributions, and can distinguish in-distribution samples from out-of-distribution samples to a certain extent [29,30]. Based on this property, this paper uses an energy threshold to construct a reliable pseudo-label selection mask. Specifically, given an energy threshold $\lambda_{e}$, a pixel is regarded as a reliable prediction and retained for subsequent training only when its energy score is lower than the threshold. The corresponding selection mask is defined as

(4) $$M^{\left(h,w\right)}=\left\{\begin{array}{cc} 1, & \mathrm{if}\;E_{X_{rf}}^{\left(h,w\right)}\leq \lambda_{e},\\ 0, & \mathrm{otherwise}. \end{array}\right.$$

where $M^{\left(h,w\right)}\in \left\{0,1\right\}$ indicates whether the pixel location, (h,w) is selected as a valid pseudo-label, and $\lambda_{e}$ denotes the predefined threshold. Through this process, target-domain supervision is restricted to relatively reliable prediction regions, thereby reducing noise interference caused by high-confidence incorrect predictions.

Although the energy-threshold-based selection strategy can improve pseudo-label quality, relying only on static filtering is still insufficient to fully suppress pseudo-label noise in the early training stage. Since the model has not yet formed stable target-domain decision boundaries at the beginning of training, the retained pseudo-labels may still contain errors. Directly assigning them a fixed weight may cause noisy supervision to accumulate and propagate. Therefore, we adopt a training stage-dependent weight $\beta_{t}$ to gradually strengthen target-domain supervision as training progresses.

Based on this dynamic weighting strategy, the final pseudo-label weight map is defined as:

(5) $$W^{\left(h,w\right)}=\mathrm{clip}\left(\beta_{t}\cdot M^{\left(h,w\right)},\;0,\;1\right),$$

where $M^{\left(h,w\right)}$ denotes the energy-based selection mask, and $\beta_{t}$ denotes the dynamic weight for pseudo-label supervision at iteration *t*. In practice, $\beta_{t}$ is assigned a relatively small value in the early training stage, gradually increased during the intermediate stage, and maintained at a maximum value in the later stage. This dynamic weight adjustment gradually increases the contribution of target-domain pseudo-label supervision to the optimization objective, thereby reducing the risk of noisy supervision accumulation in the early stage. The function clip(·) ensures that the weight value remains within the range [0,1].

Based on the above pseudo-label selection and weight adjustment strategy, the weighted cross-entropy loss for the target domain is defined as

(6) $$L_{dl}=\frac{1}{HW}\sum_{h=1}^{H}\sum_{w=1}^{W}W^{\left(h,w\right)}\ell_{ce}\left(z_{rf}^{\left(h,w\right)},\;{\hat{Y}}_{rf}^{\left(h,w\right)}\right).$$

where ${\hat{Y}}_{rf}^{\left(h,w\right)}$ denotes the class label corresponding to pixel (h,w) in the pseudo-label map ${\hat{Y}}_{rf}$, $z_{rf}^{\left(h,w\right)}$ denotes the *C*-dimensional logits vector output by network $S_{\varphi }$ at this pixel location, $W^{\left(h,w\right)}$ denotes the pseudo-label weight map obtained from Equation (5), and $\ell_{ce}\left(\cdot \right)$ denotes the pixel-level cross-entropy loss.

To verify the effect of DEPL on pseudo-label quality, this paper compares three pseudo-label strategies on the ACDC-Fog dataset: confidence score-based selection, static energy selection, and DEPL, using correct ratio and incorrect ratio as evaluation metrics. Among them, DEPL-ND denotes the variant that only uses static energy selection without dynamic weight adjustment. As shown in Table 1, the confidence score-based selection method still retains many incorrect pseudo-labels, with a correct ratio of 91.5% and an incorrect ratio of 8.5%. In contrast, DEPL-ND increases the correct ratio to 92.1% and reduces the incorrect ratio to 7.9%, indicating that energy scores can select reliable predictions more effectively than confidence scores. After further introducing dynamic weight adjustment, DEPL increases the correct ratio to 93.8% and reduces the incorrect ratio to 6.2%. These results show that DEPL can increase the proportion of reliable pseudo-labels while reducing the retention of incorrect pseudo-labels, thereby providing more stable supervision signals for target-domain self-training.

**Figure 3:**
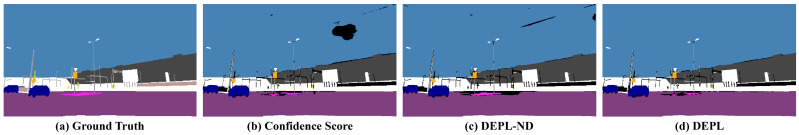
Pixel-level visualization of pseudo-label quality under different selection strategies on the ACDC-Fog dataset. (**a**) Ground truth. (**b**) Confidence score-based selection. (**c**) DEPL-ND with static energy selection. (**d**) The proposed DEPL. Colored regions indicate correct semantic predictions, while black regions indicate incorrect pseudo-labels.

In addition to quantitative metrics, this paper further observes the influence of different pseudo-label selection strategies on the retention of erroneous pseudo-labels through pixel-level visualization results. As shown in Figure 3, the Confidence Score method still retains many black erroneous regions in the target domain, indicating that relying solely on confidence-based selection can easily preserve some incorrect pseudo-labels. Compared with the Confidence Score, DEPL-ND reduces part of the erroneous regions, demonstrating that static energy selection can improve pseudo-label reliability. After further introducing DEPL, the black erroneous regions are significantly reduced, and the target-domain pseudo-labels become more consistent with the ground truth, indicating that dynamic energy-guided pseudo-labeling can effectively reduce the interference of erroneous supervision in target-domain training.

### 3.3. Reliable-Region Class Confusion Calibration

Although DEPL can reduce the interference of noisy pseudo-labels in target-domain self-training, its main role is to select reliable supervision signals, and it cannot directly constrain unreasonable overlaps among the prediction distributions of different classes. To further improve the class discriminability of target-domain predictions, this paper proposes the Reliable-region Class Confusion Calibration (RCC) module, which calibrates class relationships within reliable prediction regions and enhances the discriminative ability of target-domain predictions.

When an unlabeled target-domain foggy image $X_{rf}$ is fed into the network $S_{\varphi }$, the network outputs the corresponding pixel-level logits map $z_{rf}=S_{\varphi }\left(X_{rf}\right)\in R^{B\times C\times H\times W}$. After applying the softmax operation, the pixel-level class probability map is obtained as

(7) $$N=softmax\left(z_{rf}\right)\in R^{B\times C\times H\times W},$$

where *B*, *C*, *H*, and *W* denote the batch size, the number of classes, the image height, and the image width, respectively. Considering that target-domain predictions may still contain unreliable regions, this paper models class relationships only within reliable pixel regions. To this end, the pseudo-label weight map $W^{\left(h,w\right)}$ generated by DEPL in Equation (5) is used to weight and filter the class probability map, producing the weighted target-domain probability map as follows:

(8) $$\tilde{N}=N⊙\tilde{Z},\;\tilde{Z}\in R^{B\times 1\times H\times W},$$

where ⊙ denotes element-wise multiplication, and $\tilde{Z}=W^{\left(h,w\right)}$. Through this operation, the subsequent class relationship modeling is restricted to reliable target-domain regions, thereby reducing the interference of noisy predictions on class constraints. Therefore, RCC is not a class constraint independent of DEPL, but is region-guided by the reliability prediction results of DEPL.

On this basis, the weighted probability map is unfolded along the spatial dimension as G=H×W, namely ${\tilde{N}}_{b}\in R^{C\times G}$. Since the number of pixels belonging to different classes usually varies significantly in an image, directly computing class relationships based on the original probability distributions may cause large-area categories to dominate the statistics, thereby affecting the characterization of relationships among different classes. Therefore, this paper normalizes each class channel along the spatial dimension, so that the probability responses of different class channels are mapped to a relatively unified scale, reducing the influence of category area differences on confusion modeling as follows:

(9) $${\hat{N}}_{b}\left(i,k\right)=\frac{{\tilde{N}}_{b}\left(i,k\right)}{\sum_{k^{\prime }=1}^{G}{\tilde{N}}_{b}\left(i,k^{\prime }\right)+\epsilon },\;i=\left(1,\ldots ,C\right),\;k=\left(1,\ldots ,G\right),$$

where *i* denotes the class index, *k* denotes the current pixel index, and *b* denotes the index of the *b*-th sample in the batch; ϵ is a small constant for numerical stability. ${\tilde{N}}_{b}\left(i,k\right)$ denotes the predicted probability of class *i* for the *k*-th pixel in the *b*-th sample, and $\sum_{k^{\prime }=1}^{G}{\tilde{N}}_{b}\left(i,k^{\prime }\right)$ denotes the probability sum of class *i* over the whole image. After Equation (9), the probability response of each class channel is re-normalized along the spatial dimension, thereby reducing the influence of category area differences on class relationship estimation and improving the comparability and numerical stability of class confusion matrix calculation.

**Figure 4:**
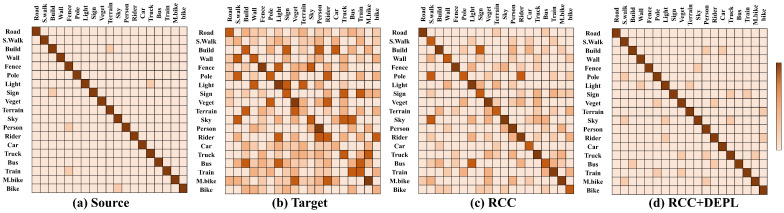
Normalized class confusion matrices of different prediction results. (**a**) Source-domain prediction. (**b**) Target-domain prediction. (**c**) RCC applied to the entire target-domain prediction map. (**d**) RCC+DEPL applied within reliable prediction regions. Darker colors indicate stronger class correlations. Diagonal responses reflect intra-class consistency, while off-diagonal responses reflect inter-class confusion.

Subsequently, the class confusion matrix is constructed based on the normalized probability distribution as follows:

(10) $$C_{b}={\hat{N}}_{b}{\hat{N}}_{b}^{T}\in R^{C\times C},$$

where $C_{b}$ denotes the class confusion matrix of the *b*-th sample. The diagonal responses of the matrix reflect intra-class consistency, while the off-diagonal responses reflect the degree of inter-class confusion. As shown in Figure 4, the responses of Source in Figure 4a are mainly concentrated in the diagonal regions, while the off-diagonal regions show lighter colors, indicating that source-domain predictions have better class discriminability. In contrast, the off-diagonal regions of Target in Figure 4b contain more dark responses, suggesting that the prediction distributions of different classes in the real foggy target domain overlap more significantly and exhibit a higher degree of class confusion. After introducing RCC, as shown in Figure 4c, the dark responses in the off-diagonal regions are clearly reduced, indicating that RCC can suppress unreasonable correlations between different classes and reduce inter-class prediction overlap. It should be noted that Figure 4 is only used to analyze the effect of the RCC module on the class confusion matrix when it is applied independently. In this case, RCC is directly applied to the entire target-domain prediction map.

Based on the above analysis, this paper uses the proportion of off-diagonal elements to measure the degree of class confusion. The loss is defined as

(11) $$L_{rcc}=\frac{1}{B}\sum_{b=1}^{B}\frac{\sum_{i\neq j}C_{b}\left(i,j\right)}{\sum_{i,j}C_{b}\left(i,j\right)+\epsilon }.$$

The numerator $\sum_{i\neq j}C_{b}\left(i,j\right)$ denotes the sum of all off-diagonal elements, which characterizes the overall degree of inter-class confusion, while the denominator $\sum_{i,j}C_{b}\left(i,j\right)$ denotes the sum of all elements in the matrix for normalization. By minimizing Equation (11), the model reduces the proportion of off-diagonal confusion responses in the overall class relationship, thereby alleviating inter-class confusion in target-domain predictions. Here, the off-diagonal responses quantify the overlap of different class probability distributions at the same spatial locations, rather than the normal contextual co-occurrence of different categories.

**Figure 5:**
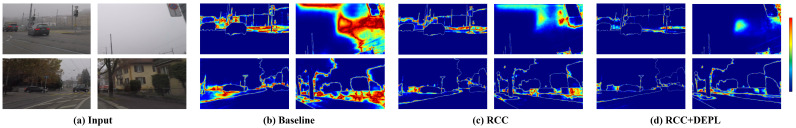
Visualization results of class confusion on target-domain foggy images. (**a**) Input images. (**b**) Baseline. (**c**) RCC applied to the entire target-domain prediction map. (**d**) RCC+DEPL applied within reliable prediction regions. Red and yellow regions indicate higher class confusion, while blue regions indicate lower class confusion.

To further observe the effect of RCC on class confusion at the pixel level, Figure 5 presents the visualization results of class confusion on target-domain foggy images. It can be observed that the Baseline produces many red and yellow high-response regions around road–vehicle boundaries, building contours, and distant low-contrast areas, indicating that the class prediction distributions in these regions have obvious overlaps. After introducing RCC, the high-confusion response regions are significantly reduced, and the responses become more concentrated near target boundaries, indicating that RCC can reduce local class confusion in the foggy target domain and improve the class discrimination ability in boundary and degraded regions. It should be noted that Figure 5 is only used to analyze the effect of the RCC module on class confusion visualization when it is applied independently. In this case, RCC is directly applied to the entire target-domain prediction map.

Combining DEPL and RCC, the total loss function of this paper is defined as

(12) $$L_{total}=L_{sup}+\lambda_{dl}L_{dl}+\lambda_{rcc}L_{rcc}.$$

where $\lambda_{dl}$ and $\lambda_{rcc}$ denote the weight coefficients of the dynamic energy-guided pseudo-labeling loss and the reliable-region class confusion calibration loss, respectively. These two terms optimize the target-domain learning process from the perspectives of supervision signal reliability and class relationship constraints, jointly improving the model’s adaptability to real foggy scenes.

## 4. Experiments

### 4.1. Datasets

We evaluate the proposed method on several real foggy and adverse-weather scene datasets. Cityscapes [21] is used as the source-domain dataset, which contains 3450 finely annotated urban street scene images collected from 50 cities, including 2975 training images and 500 validation images, with a resolution of 2048×1024. Foggy Zurich [31] is used as the unlabeled target-domain dataset for unsupervised domain adaptation training, containing 3808 real foggy urban street scene images with a resolution of 1920×1080 collected in and around Zurich. Foggy Driving [32] contains 101 real foggy urban street scene images and is only used for qualitative and quantitative evaluation. ACDC [33] contains real urban street scene images under various adverse weather conditions. We use the foggy, rainy, and snowy samples from its validation set, where the foggy samples are used to evaluate segmentation performance in foggy scenes, and the rainy and snowy samples are used to evaluate cross-weather generalization ability.

### 4.2. Implementation Details

Following FIFO [23], we adopt RefineNet-lw [14] with a ResNet-101 [19] backbone as the basic segmentation framework, initialized with model parameters pretrained on Cityscapes. The network is trained using stochastic gradient descent (SGD), with momentum of 0.9 and weight decay of $1\times 10^{-5}$. The initial learning rates of the encoder and decoder are set to $6\times 10^{-4}$ and $6\times 10^{-3}$, respectively, and a polynomial learning rate decay strategy with a power of 0.9 is adopted. During training, random scaling, 600×600 random cropping, and random horizontal flipping are used for data augmentation. Gradient accumulation and gradient clipping are applied to improve training stability.

For hyperparameter settings, the energy selection threshold $\lambda_{e}$ is set to −15, the dynamic weight $\beta_{t}$ is initialized to 0.8 and increased to a maximum value of 1, $\lambda_{dl}$ is set to 1, $\lambda_{rcc}$ is set to 0.05, and the EMA update coefficient is set to 0.999. All experiments are conducted on a single NVIDIA RTX 3090 GPU (NVIDIA Corporation, Santa Clara, CA, USA). To ensure reliable results, we evaluate the model using three random seeds and report the average results. The mean Intersection over Union (mIoU) is used as the evaluation metric.

### 4.3. Compared Methods

To comprehensively verify the effectiveness of the proposed method, RPCC is compared with several representative unsupervised domain-adaptive semantic segmentation methods. RefineNet-lw [14] trained only on Cityscapes is used as the baseline model to measure the performance without target-domain adaptation. The compared methods include conventional UDA methods, namely AdvEnt [2] and FDA [3]; image-to-image translation methods, namely CycleGAN [34], CUT [35], and StyTr2 [36]; Transformer-based UDA methods, namely HRDA [37] and BWG [38]; and foggy-scene-specific domain adaptation methods, namely CMAda3+ [20], FIFO [23], CuDA-Net+ [24], TDo-Dif [4], SCM [39], and DAEN [25].

It should be noted that the results in Table 2 and Table 3 are obtained from two sources. Methods marked with † were reproduced and evaluated by us under the experimental setting described in Section 4.2, whereas methods marked with ‡ were directly collected from the corresponding publications. Since the latter may differ in backbone networks, input resolutions, training data, synthetic or intermediate domains, and adaptation procedures, they are included as reference results rather than strictly controlled comparisons. Therefore, the main controlled comparison in this work is based on the reproduced methods, the proposed RPCC, and the ablation variants evaluated under our experimental setting.

### 4.4. Comparison with Other UDA Methods

#### 4.4.1. Quantitative Comparison

Table 2 and Table 3 show the quantitative comparison results on the Foggy Driving dataset [32] and the ACDC-Fog dataset [33]. It can be observed that the proposed RPCC outperforms all comparison methods on both test sets, demonstrating its effectiveness for semantic segmentation in real foggy scenes.

On the Foggy Driving dataset shown in Table 2, RPCC achieves consistent improvements over conventional UDA methods and image-to-image translation (I2I) domain adaptation methods. Compared with foggy-scene-specific UDA methods, RPCC improves the mIoU by 6.2%, 5.3%, 2.6%, 9.2%, 3.0%, and 2.5% over CMAda3+, FIFO, CuDA-Net+, TDo-Dif, DAEN, and SCM, respectively. In addition, compared with recent Transformer-based UDA methods, RPCC also achieves further improvements, indicating that the proposed method not only has clear advantages in foggy-scene domain adaptation but also remains competitive with strong cross-domain semantic segmentation models.

On the ACDC-Fog dataset shown in Table 3, RPCC improves the mIoU by 17.7%, 1.6%, 1.4%, and 2.8% over FIFO, TDo-Dif, DAEN, and SCM, respectively. These results further indicate that RPCC maintains strong segmentation performance on real foggy scenes in another adverse-weather dataset. Further class-level analysis shows that RPCC performs notably better than the comparison methods on key categories such as road, building, car, truck, and bus. Beyond the overall mIoU gains, the improved recognition of road regions and traffic participants is practically relevant for reliable scene understanding under degraded visibility, as these categories are closely associated with drivable-area perception and traffic-aware decision-making. The qualitative results further show more coherent road predictions, more complete vehicle regions, and clearer object boundaries in foggy scenes.

#### 4.4.2. Qualitative Comparison

**Figure 6:**
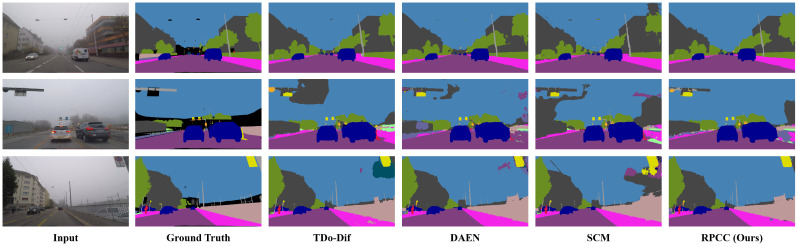
Qualitative comparison between the proposed RPCC and existing mainstream methods on the Foggy Driving dataset. From left to right: input image, ground truth, TDo-Dif, DAEN, SCM, and RPCC.

**Figure 7:**
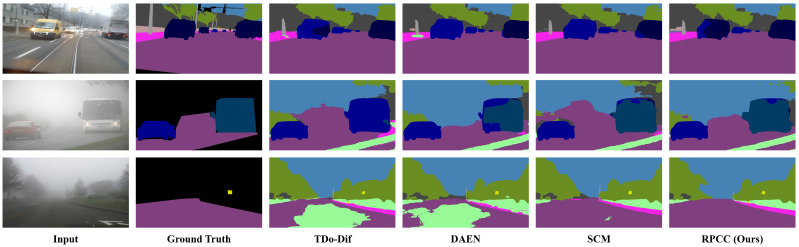
Qualitative comparison between the proposed RPCC and existing mainstream methods on the ACDC-Fog dataset. From left to right: input image, ground truth, TDo-Dif, DAEN, SCM, and RPCC.

We further conduct qualitative comparisons with TDo-Dif, DAEN, and SCM on the Foggy Driving and ACDC-Fog datasets. As shown in Figure 6 and Figure 7, the comparison methods still suffer from incomplete object segmentation and local class confusion in real foggy scenes, especially around vehicles, road boundaries, and distant low-contrast regions. In contrast, RPCC produces more coherent predictions for road regions and more accurate segmentation results for traffic participants such as cars and trucks. Moreover, RPCC preserves clearer object boundaries in blurred areas and reduces incorrect predictions between visually similar categories. These qualitative results indicate that the proposed method not only improves the overall segmentation accuracy but also effectively alleviates class confusion in foggy scenes.

### 4.5. Ablation Study and Parameter Sensitivity Analysis

#### 4.5.1. Statistical Stability Analysis

To evaluate the stability of the methods reproduced under our experimental setting, we repeated the training of each method with three random seeds and report the mean and standard deviation of the overall mIoU. Methods whose results were directly collected from the corresponding publications are not included in this analysis because seed-wise results are unavailable. The results are summarized in Table 4.

As shown in Table 4, RPCC achieves the highest mean mIoU on both datasets while maintaining low standard deviations across different random seeds. These results demonstrate that the performance improvement of RPCC is stable across independent runs and is not caused by random initialization.

#### 4.5.2. Effectiveness Analysis of DEPL and RCC

We first conduct ablation experiments on the Foggy Driving dataset to verify the effectiveness of the key designs in the proposed framework. The results are shown in Table 5. The Baseline is trained only on the clear-weather source domain and does not use foggy target-domain images for adaptive optimization. Therefore, its segmentation performance in real foggy scenes is limited, achieving only 43.6% mIoU, which indicates an obvious domain shift problem.

After introducing confidence score-based pseudo-label supervision on the basis of the Baseline, the model performance improves to 48.3%, which is 4.7% higher than the Baseline. This indicates that target-domain pseudo-label self-training can alleviate the distribution shift from the source domain to the real foggy target domain to a certain extent. However, confidence scores tend to retain high-confidence incorrect predictions, so the performance improvement remains limited. In contrast, after introducing DEPL, the model performance further improves to 51.3%, achieving a 7.7% improvement over the Baseline. This result demonstrates that energy score-based pseudo-label reliability modeling can provide more stable target-domain supervision than simple confidence-based selection.

When RCC is introduced alone on the basis of the Baseline, the model performance improves to 50.1%, which is 6.5% higher than the Baseline. Here, RCC denotes the class confusion calibration module directly applied to the entire target-domain prediction map. This result indicates that even without pseudo-label supervision, class confusion calibration can alleviate class prediction overlap in foggy scenes to a certain extent and improve the discriminability of target-domain predictions.

When DEPL and RCC are introduced simultaneously, the complete RPCC achieves the best performance, with the mIoU further increasing to 56.0%. Compared with using DEPL and RCC alone, the complete method improves the mIoU by 4.7% and 5.9%, respectively. These results show that both dynamic energy-guided pseudo-label selection and reliable-region class confusion calibration can effectively improve the segmentation performance in real foggy target domains.

In addition, Table 5 further compares the effects of different pseudo-label strategies combined with RCC. When confidence-based selection is combined with RCC, the model achieves 53.3% mIoU. When DEPL-ND, which only uses static energy selection without dynamic weight adjustment, is combined with RCC, the performance further improves to 54.1%. The complete RPCC achieves the best result of 56.0%. This result indicates that energy scores can provide a more reliable basis for pseudo-label selection than confidence scores, while the training-stage dynamic weight adjustment in DEPL further improves the stability of pseudo-label supervision.

For the class confusion calibration strategy, when RCC is directly applied to the entire target-domain prediction map, the combination of DEPL and RCC achieves 54.4% mIoU. This indicates that class confusion calibration can alleviate class prediction overlap in the target domain, but it may still be affected by unreliable prediction regions. When class channel normalization is removed from RCC, the combination of DEPL and RCC achieves 55.3% mIoU, indicating that modeling class confusion within reliable regions can further reduce the interference of noisy predictions on confusion statistics. Finally, the complete RPCC achieves the best result of 56.0%, demonstrating that class channel normalization can further reduce the influence of class area differences on confusion statistics under reliable-region constraints, thereby improving the stability of class relationship estimation.

#### 4.5.3. Sensitivity Analysis of the EMA Update Ratio and Loss Weights

Table 6 reports the sensitivity analysis results of the EMA update ratio α, the RCC loss weight $\lambda_{rcc}$, and the pseudo-label supervision loss weight $\lambda_{dl}$ on the Foggy Driving dataset. It can be observed that the model achieves the best performance when α=0.999, and thus the EMA update ratio of the momentum teacher network is set to 0.999. For the RCC loss weight, the model obtains the best result when $\lambda_{rcc}=0.05$. When $\lambda_{rcc}$ is too large or too small, the model performance decreases, indicating that an appropriate $\lambda_{rcc}$ can achieve a better balance between class relationship regularization and main-task learning. For the pseudo-label supervision loss weight, the model performs best when $\lambda_{dl}=1.0$, while smaller or larger values lead to performance degradation. Therefore, $\lambda_{rcc}$ and $\lambda_{dl}$ are finally set to 0.05 and 1.0, respectively.

#### 4.5.4. Sensitivity Analysis of Energy Threshold and Dynamic Pseudo-Label Selection Weight

We further analyze the influence of the energy threshold $\lambda_{e}$ and the dynamic pseudo-label selection weight $\beta_{t}$ in DEPL. As shown in Table 7, different energy thresholds significantly affect the quality of pseudo-label selection. When $\lambda_{e}=-15$, the model achieves the best performance on the Foggy Driving dataset. Lower thresholds, such as −19 and −17, are too strict and limit the number of target-domain pixels available for self-training, making it difficult to sufficiently learn target-domain knowledge. In contrast, higher thresholds, such as −13 and −11, introduce more noisy pseudo-labels and lead to performance degradation. Therefore, $\lambda_{e}=-15$ achieves the best balance between reliable pseudo-label selection and effective target-domain adaptation.

Table 7 also reports the influence of the dynamic pseudo-label selection weight $\beta_{t}$. It can be observed that the model achieves the best result when $\beta_{t}=0.8$. When $\beta_{t}$ is further increased or decreased, the performance declines. This indicates that an appropriate dynamic weight can more effectively regulate the strength of target-domain supervision, thereby improving the stability of self-training while suppressing noise propagation. Therefore, $\lambda_{e}$ and $\beta_{t}$ are finally set to −15 and 0.8, respectively.

### 4.6. Computational Overhead Analysis

To further analyze the computational overhead of the proposed RPCC, we compare it with the RefineNet-LW baseline and two representative foggy-scene adaptation methods, FIFO and TDo-Dif. All measurements are conducted under the same experimental environment with an input resolution of 1920×1080.

As shown in Table 8, RPCC preserves the lightweight inference property of the RefineNet-LW baseline. Both methods contain 46.35 M parameters and require 410.33 GMACs, while their inference latency is nearly identical at 48.22 ms/image and 48.37 ms/image, respectively. Compared with TDo-Dif, RPCC reduces the training time, GPU memory, parameter count, computational complexity, and inference latency by 37.0%, 13.1%, 60.7%, 80.3%, and 63.1%, respectively. RPCC also requires lower training overhead and inference cost than FIFO, which uses additional FogPassFilter modules and a three-scale inference protocol. Overall, RPCC achieves a favorable balance between segmentation performance and computational efficiency.

### 4.7. Generalization Experiment

To further verify the cross-weather robustness of the proposed method, this paper extends the test scenarios to the rainy and snowy subsets of the ACDC dataset while keeping the training configuration of the foggy-scene domain adaptation task unchanged. It should be noted that, in this experiment, the model does not access any labeled or unlabeled data from rainy or snowy scenes during training. Therefore, this experiment aims to evaluate the generalization ability of the model under unseen adverse weather conditions.

The proposed RPCC is compared with four recent representative methods, namely FIFO, TDo-Dif, DAEN, and SCM, and the quantitative results are shown in Table 9. It can be observed that although the training process only relies on foggy target-domain data, RPCC still achieves better segmentation performance under both unseen rainy and snowy conditions. Specifically, compared with SCM, RPCC improves the mIoU by 1.7% and 1.5% on rainy and snowy scenes, respectively; compared with DAEN, RPCC improves the mIoU by 4.1% and 1.8%, respectively; compared with TDo-Dif, the improvements are 7.3% and 2.8%, respectively; compared with FIFO, the improvements are 11.1% and 6.1%, respectively. These results indicate that the proposed confusion-aware self-training framework not only improves target-domain adaptation performance in foggy scenes but also exhibits certain cross-weather robustness.

#### Qualitative Comparison Under Cross-Weather Conditions

**Figure 8:**
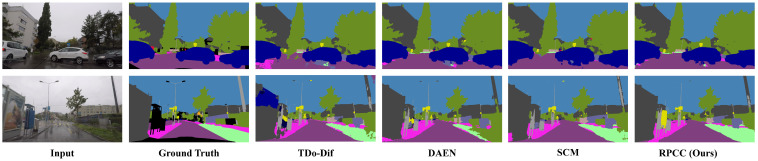
Qualitative comparison among RPCC, TDo-Dif, DAEN, and SCM on the ACDC-Rain dataset. From left to right: input image, ground truth, TDo-Dif, DAEN, SCM, and RPCC.

**Figure 9:**
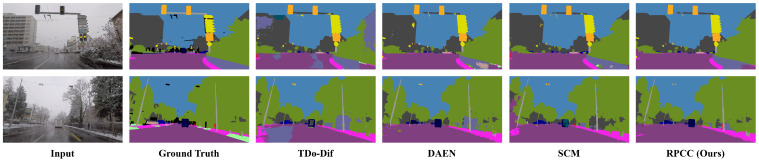
Qualitative comparison among RPCC, TDo-Dif, DAEN, and SCM on the ACDC-Snow dataset. From left to right: input image, ground truth, TDo-Dif, DAEN, SCM, and RPCC.

We further conduct qualitative comparisons among RPCC, TDo-Dif, DAEN, and SCM on the rainy and snowy subsets of the ACDC dataset. As shown in Figure 8 and Figure 9, RPCC produces overall better segmentation results than the comparison methods in rainy and snowy scenes that are not involved in training. In particular, RPCC provides more stable semantic predictions in road regions, vehicle objects, and local areas severely affected by weather degradation, while reducing local class confusion. This indicates that the proposed method can maintain certain adaptability to other adverse weather scenarios even when it is trained only with foggy target-domain data.

## 5. Discussion

The experimental results indicate that the performance gains of RPCC arise from the complementary roles of reliable pseudo-labeling and class confusion calibration. Compared with confidence-based pseudo-label selection, DEPL uses energy scores to identify more reliable target-domain predictions and reduces the retention of high-confidence incorrect pseudo-labels. RCC further calibrates class relationships within reliable prediction regions to suppress unreasonable overlaps among different class prediction distributions. Therefore, RPCC not only improves the quality of pseudo-label supervision but also alleviates class confusion caused by degraded visual information in real foggy scenes.

The ablation results further demonstrate the benefit of combining DEPL and RCC. When DEPL is used alone, the model benefits from more reliable target-domain supervision, but class prediction overlap is not explicitly constrained. When RCC is used alone, class confusion can be alleviated to a certain extent, but the calibration process may still be affected by unreliable target-domain predictions. By applying RCC within the reliable regions identified by DEPL, the proposed framework reduces the influence of noisy predictions on class relationship modeling and improves the stability of confusion calibration. This explains why the complete RPCC achieves better performance than either DEPL or RCC used independently.

The cross-weather evaluation results also indicate that RPCC exhibits some robustness under unseen adverse weather conditions. Although the model is trained only with foggy target-domain data, it still achieves improved performance on rainy and snowy scenes. This suggests that reliable pseudo-label supervision and class relationship calibration can help maintain more stable semantic predictions under other degraded visual conditions. However, the current framework still relies on manually selected hyperparameters, such as the energy threshold and loss weights. In addition, RCC models class relationships primarily based on prediction probabilities, while more explicit structural cues and temporal information are not fully explored. Future work will investigate adaptive threshold selection strategies and stronger structure-aware calibration mechanisms to further improve robustness in diverse adverse weather scenarios.

## 6. Conclusions

In this paper, we propose RPCC, a reliable pseudo-labeling and confusion calibration self-training framework for semantic segmentation in real foggy scenes. RPCC jointly addresses pseudo-label noise and class confusion in target-domain learning through dynamic energy-guided pseudo-labeling (DEPL) and reliable-region class confusion calibration (RCC).

Experiments on real foggy-scene datasets demonstrate that RPCC achieves strong quantitative and qualitative segmentation performance compared with existing UDA methods. Further evaluations on unseen rainy and snowy scenes show that RPCC maintains favorable cross-weather generalization. These results demonstrate that jointly improving pseudo-label reliability and reducing class confusion is effective for robust semantic segmentation under degraded visual conditions.

## Figures and Tables

**Table 1:** Comparison of correct and incorrect ratios under different pseudo-label selection strategies on the ACDC-Fog dataset. DEPL-ND denotes the variant that only uses static energy selection without dynamic weight adjustment.

Pseudo-Label Strategy	Correct Ratio (%) ↑	Incorrect Ratio (%) ↓
Confidence Score	91.5	8.5
DEPL-ND	92.1	7.9
DEPL	93.8	6.2

**Table 2:** Comparison of mIoU (%) between the proposed RPCC and existing mainstream methods on the Foggy Driving dataset. The best and second-best results are highlighted in bold and underlined, respectively. Methods marked with † are reproduced by us, whereas methods marked with ‡ are directly collected from the corresponding publications.

Method	Road	S.Walk	Build.	Wall	Fence	Pole	Tr.Light	Tr.Sign	Veget.	Terrain	Sky	Person	Rider	Car	Truck	Bus	Train	M.Bike	Bike	mIoU
^†^ Baseline	88.0	26.6	68.1	28.5	14.6	42.5	44.3	54.5	63.0	9.1	86.9	64.5	46.7	65.0	6.8	13.0	27.5	28.7	49.2	43.6
^‡^ AdSegNet	–	–	–	–	–	–	–	–	–	–	–	–	–	–	–	–	–	–	–	29.7
^‡^ AdvEnt	–	–	–	–	–	–	–	–	–	–	–	–	–	–	–	–	–	–	–	46.8
^‡^ FDA	–	–	–	–	–	–	–	–	–	–	–	–	–	–	–	–	–	–	–	21.8
^†^ CycleGAN	92.2	35.3	68.1	27.4	14.1	46.7	49.8	58.7	71.2	11.9	88.1	66.0	41.0	66.8	7.0	19.1	60.9	30.8	53.0	47.8
^†^ CUT	92.1	34.2	68.2	20.8	13.8	46.5	49.2	57.0	70.2	12.5	87.6	66.2	41.3	66.9	9.8	16.9	61.3	30.5	52.1	47.2
^†^ StyTr2	91.8	33.7	66.6	**36.2**	10.5	33.0	46.4	53.0	71.7	7.3	87.6	66.3	46.1	65.4	18.1	11.6	33.8	35.0	50.6	45.5
^‡^ HRDA	–	–	–	–	–	–	–	–	–	–	–	–	–	–	–	–	–	–	–	46.6
^‡^ BWG	–	–	–	–	–	–	–	–	–	–	–	–	–	–	–	–	–	–	–	54.2
^‡^ CMAda3+	–	–	–	–	–	–	–	–	–	–	–	–	–	–	–	–	–	–	–	49.8
^†^ FIFO	90.8	39.1	72.9	24.2	20.0	42.3	51.0	59.1	72.0	9.4	90.2	64.7	48.5	71.0	25.4	65.8	43.4	24.8	49.1	50.7
^‡^ CuDA-Net+	–	–	–	–	–	–	–	–	–	–	–	–	–	–	–	–	–	–	–	53.4
^†^ TDo-Dif	91.7	33.0	72.6	22.7	**21.1**	43.1	46.1	57.2	70.9	11.4	87.3	66.1	41.1	72.3	37.8	15.5	30.0	25.0	44.2	46.8
^†^ DAEN	92.5	**42.6**	70.6	27.8	20.6	45.5	50.6	**60.1**	73.4	11.4	**90.4**	69.1	**51.9**	70.4	16.1	62.9	65.1	33.3	53.5	53.0
^†^ SCM	92.1	38.4	66.7	35.9	17.9	**48.2**	49.9	57.5	70.5	13.0	86.2	66.2	50.0	70.4	23.3	67.6	**77.2**	35.6	49.9	53.5
RPCC (Ours)	**92.6**	31.8	**75.1**	26.5	19.2	45.2	**51.1**	58.6	**73.5**	**17.2**	88.9	**70.4**	47.3	**77.1**	**59.4**	**77.6**	61.8	**36.5**	**54.2**	**56.0**

**Table 3:** Comparison of mIoU (%) between the proposed RPCC and existing mainstream methods on the ACDC-Fog dataset. The best and second-best results are highlighted in bold and underlined, respectively. Methods marked with † are reproduced by us.

Method	Road	S.Walk	Build.	Wall	Fence	Pole	Tr.Light	Tr.Sign	Veget.	Terrain	Sky	Person	Rider	Car	Truck	Bus	Train	M.Bike	Bike	mIoU
^†^ Baseline	79.8	65.2	67.8	18.7	26.5	30.5	59.8	54.7	71.2	49.2	93.6	41.6	49.8	69.9	20.9	25.2	79.3	12.1	56.4	51.2
^†^ CycleGAN	94.9	75.5	74.0	36.7	32.7	37.3	65.6	62.3	82.6	56.1	94.3	46.9	57.0	83.6	43.2	60.0	78.7	40.1	60.0	62.2
^†^ CUT	94.4	74.1	76.2	41.1	32.2	36.8	65.4	61.5	82.6	54.4	96.0	43.0	58.0	83.3	46.6	71.0	82.4	59.0	57.2	64.0
^†^ StyTr2	87.0	73.8	72.5	42.9	28.9	31.9	61.1	54.1	77.9	52.5	85.5	**49.2**	51.0	80.0	30.1	45.5	45.3	33.6	59.6	55.8
^†^ FIFO	51.3	64.9	71.2	22.0	26.8	27.7	49.2	52.3	73.2	46.1	60.3	48.5	52.5	74.8	16.9	54.1	67.4	16.2	60.6	49.3
^†^ TDo-Dif	94.3	73.8	82.3	**50.7**	**44.3**	44.8	60.1	59.0	**85.9**	46.3	95.1	42.1	**65.9**	84.6	48.3	70.6	87.6	**66.3**	41.2	65.4
^†^ DAEN	95.5	81.4	**82.7**	48.2	35.5	44.4	68.8	**66.5**	85.7	55.3	**97.3**	46.7	58.3	85.6	41.5	74.2	88.3	27.8	61.7	65.6
^†^ SCM	92.6	80.9	73.9	47.9	33.0	**48.9**	70.4	57.7	83.9	**57.4**	92.2	50.4	62.6	82.9	**56.1**	40.7	79.4	45.6	**63.3**	64.2
RPCC (Ours)	**96.5**	**82.9**	78.7	46.4	33.0	46.0	**70.9**	56.3	84.9	53.3	96.0	44.9	60.4	**87.1**	45.1	**81.3**	**91.0**	59.9	59.0	**67.0**

**Table 4:** Statistical comparison of overall mIoU (%) for methods reproduced under our experimental setting over three random seeds on the Foggy Driving and ACDC-Fog datasets. Results are reported as mean ± standard deviation. The best results are highlighted in bold.

Method	Foggy Driving	ACDC-Fog
Baseline	43.6 ± 0.31	51.2 ± 0.28
CycleGAN	47.8 ± 0.35	62.2 ± 0.41
CUT	47.2 ± 0.38	64.0 ± 0.33
StyTr2	45.5 ± 0.44	55.8 ± 0.47
FIFO	50.7 ± 0.32	49.3 ± 0.46
TDo-Dif	46.8 ± 0.41	65.4 ± 0.34
DAEN	53.0 ± 0.26	65.6 ± 0.29
SCM	53.5 ± 0.30	64.2 ± 0.37
RPCC (Ours)	**56.0 ± 0.22**	**67.0 ± 0.24**

**Table 5:** Comprehensive ablation study of the proposed RPCC framework on the Foggy Driving dataset. FD denotes the Foggy Driving dataset. The best results are highlighted in bold.

Method/Variant	Pseudo-Label Strategy	Class Confusion Mitigation Strategy	mIoU
Baseline	–	–	43.6
Baseline + Confidence	Confidence-based pseudo-labeling	–	48.3
Baseline + RCC	–	RCC on the whole target prediction	50.1
Baseline + DEPL	DEPL	–	51.3
RCC + Confidence	Confidence-based pseudo-labeling	Full RCC	53.3
RCC + DEPL-ND	Static energy-based pseudo-labeling	Full RCC	54.1
DEPL + RCC	DEPL	RCC on the whole target prediction	54.4
DEPL + RCC	DEPL	RCC without class channel normalization	55.3
RPCC (Ours)	DEPL	Full RCC	**56.0**

**Table 6:** Sensitivity analysis of EMA update ratio and loss weights on the Foggy Driving dataset. FD denotes the Foggy Driving dataset. The best results are highlighted in bold.

α	mIoU	$\lambda_{\mathrm{rcc}}$	mIoU	$\lambda_{\mathrm{dl}}$	mIoU
0.9	50.3	0.07	54.9	0.5	52.9
0.99	53.1	0.06	55.1	1.0	**56.0**
0.999	**56.0**	0.05	**56.0**	5.0	51.4
0.9999	52.3	0.04	53.9	–	–
–	–	0.03	53.8	–	–

**Table 7:** Sensitivity analysis of the energy threshold $\lambda_{e}$ and dynamic pseudo-label selection weight $\beta_{t}$ on the Foggy Driving dataset. FD denotes the Foggy Driving dataset. The best results are highlighted in bold.

$\lambda_{e}$	mIoU	$\beta_{t}$	mIoU
−19	52.2	1.0	54.0
−17	52.4	0.9	54.9
−15	**56.0**	0.8	**56.0**
−13	54.5	0.7	52.5
−11	53.8	0.6	51.2

**Table 8:** Computational overhead comparison between the proposed RPCC, the RefineNet-LW baseline, and representative competitors.

Method	Train Time (h)	GPU Memory (GiB)	Total Params (M)	GMACs	Infer. Time (ms/img)
RefineNet-LW (Baseline)	13	18.0	46.35	410.33	48.37
FIFO	33	22.8	184.62	820.20	98.74
TDo-Dif	27	23.6	118.05	2084.83	130.52
RPCC (Ours)	17	20.5	46.35	410.33	48.22

**Table 9:** Comparison of mIoU (%) between the proposed RPCC and recent representative methods on the rainy and snowy subsets of the ACDC dataset. The best results are highlighted in bold.

Method	Rain	Snow
FIFO	47.2	48.1
TDo-Dif	51.0	51.4
DAEN	54.2	52.4
SCM	56.6	52.7
RPCC (Ours)	**58.3**	**54.2**

## Data Availability

The data presented in this study are openly available in public datasets. Cityscapes is available at https://www.cityscapes-dataset.com/ (accessed on 28 June 2026); Foggy Zurich is available at https://people.ee.ethz.ch/~csakarid/Model_adaptation_SFSU_dense/ (accessed on 28 June 2026); Foggy Driving is available at https://people.ee.ethz.ch/~csakarid/SFSU_synthetic/ (accessed on 28 June 2026); and ACDC is available at https://acdc.vision.ee.ethz.ch/ (accessed on 28 June 2026).

## Abbreviations

The following abbreviations are used in this manuscript:

UDA	Unsupervised Domain Adaptation
RPCC	Reliable Pseudo-labeling and Confusion Calibration
DEPL	Dynamic Energy-guided Pseudo-Labeling
RCC	Reliable-region Class Confusion Calibration
EMA	Exponential Moving Average
mIoU	mean Intersection over Union
